# SRE-YOLOv8: An Improved UAV Object Detection Model Utilizing Swin Transformer and RE-FPN

**DOI:** 10.3390/s24123918

**Published:** 2024-06-17

**Authors:** Jun Li, Jiajie Zhang, Yanhua Shao, Feng Liu

**Affiliations:** 1 Artificial Intelligence Security Innovation Research, Beijing Information Science and Technology University, Beijing 100192, China; lijun@bistu.edu.cn (J.L.); 2022020993@bistu.edu.cn (J.Z.); 2 Department of Information Security, Beijing Information Science and Technology University, Beijing 100192, China; 3 National Computer System Engineering Research Institute of China, Beijing 100083, China; 4 School of Computer Science and Technology, East China Normal University, Shanghai 200062, China; 5 Shanghai International School of Chief Technology Officer, East China Normal University, Shanghai 200062, China

**Keywords:** deep learning, object detection, YOLOv8, Swin Transformer, feature pyramid network, computational perception

## Abstract

To tackle the intricate challenges associated with the low detection accuracy of images taken by unmanned aerial vehicles (UAVs), arising from the diverse sizes and types of objects coupled with limited feature information, we present the SRE-YOLOv8 as an advanced method. Our method enhances the YOLOv8 object detection algorithm by leveraging the Swin Transformer and a lightweight residual feature pyramid network (RE-FPN) structure. Firstly, we introduce an optimized Swin Transformer module into the backbone network to preserve ample global contextual information during feature extraction and to extract a broader spectrum of features using self-attention mechanisms. Subsequently, we integrate a Residual Feature Augmentation (RFA) module and a lightweight attention mechanism named ECA, thereby transforming the original FPN structure to RE-FPN, intensifying the network’s emphasis on critical features. Additionally, an SOD (small object detection) layer is incorporated to enhance the network’s ability to recognize the spatial information of the model, thus augmenting accuracy in detecting small objects. Finally, we employ a Dynamic Head equipped with multiple attention mechanisms in the object detection head to enhance its performance in identifying low-resolution targets amidst complex backgrounds. Experimental evaluation conducted on the VisDrone2021 dataset reveals a significant advancement, showcasing an impressive 9.2% enhancement over the original YOLOv8 algorithm.

## 1. Introduction

Assisted by advancements in artificial intelligence, unmanned aerial vehicles (UAVs) have attained rudimentary intelligent perception capabilities. Object detection, viewed through the lens of UAVs, stands as a pivotal core technology, finding widespread application across diverse domains including but not limited to traffic surveillance, power infrastructure inspection, agricultural crop analysis, and disaster response efforts [1]. However, due to factors such as elevation during flight, top-down visual capture, and broad-field lenses, UAV images often contain numerous small objects with weak features and limited information. Distinguishing between adjacent objects becomes challenging, especially in complex environments with low illumination and shadow occlusion [2,3]. Traditional detection algorithms like SIFT [4] and HOG [5] rely on manually designed feature extraction methods, capturing local features in the image to represent the target. However, they fail to capture higher-dimensional semantic information.

CNNs are widely used for various deep learning tasks due to their powerful feature extraction capabilities such as traditional data analysis methods focusing on textual data analysis and structured data analysis [6,7,8,9,10,11], as well as data architecture analysis [12] and multimedia data analysis [13]. It also has become a cornerstone in the domain of object detection, showcasing substantial advancements attributable to their unparalleled feature representation prowess, particularly accentuated in the realm of unmanned aerial vehicle (UAV) imagery analysis [14]. Object detection algorithms based on CNN, like the two-stage algorithm Faster R-CNN [15] and one-stage algorithm YOLO series [16], have demonstrated exceptional performance on image datasets containing more common targets like MS COCO and PASCAL VOC [17]. Nevertheless, when applied to UAV images, challenges arise from scale variations, sparse distributions, a high number of small objects, and a lack of satisfactory results. Moreover, the diminutive nature of objects in UAV images often results in a dearth of discernible appearance information, especially when juxtaposed with their larger counterparts, thereby amplifying the challenge of distinguishing them amidst intricate backgrounds. Furthermore, the intrinsic local modeling methodology inherent in CNNs imposes limitations on their capacity to encapsulate and integrate the broader global contextual cues prevalent in UAV imagery, further compounding the complexity of object detection in such environments [18]. Broadly speaking, the application of CNN-based object detection algorithms encounters considerable hurdles when directly transposed to the domain of UAV images, particularly in SOD tasks.

Addressing the challenges associated with SOD tasks in aerial drone imagery, we introduce a novel algorithm that builds upon the YOLOv8 model as its foundation. Firstly, to fully leverage the powerful feature extraction capabilities of a CNN and the temporal sequence processing abilities of a Transformer [19], we introduce an enhanced module utilized by Swin Transformer [20] and apply it to the CSPDarknet53 backbone network. Secondly, in light of the deficiencies observed in YOLOv8’s depth feature mapping fusion methodology, which relies on simplistic channel addition or overlay mapping techniques, causing a semantic information loss during the fusion stage, we introduce the RE-FPN structure. This involves introducing the Residual Feature Augmentation (RFA) module, utilizing a residual branch, and enhancing the original FPN structure in YOLOv8 by injecting context information from different spatial dimensions to optimize the feature representation of higher-level features [21]. Such enhancements bolster the network’s capability to effectively detect small objects. Moreover, we insert a lightweight attention mechanism, the ECA module, into the FPN structure to improve our network’s focus on crucial features, with minimal impact on network parameters and computational load. Furthermore, we incorporate a dedicated SOD network layer to further boost the performance of the model in capturing semantic information in shallower layers and utilize multi-scale features to better handle targets of different sizes. Lastly, we introduce the Dynamic Head method with multiple attention mechanisms [22] to further process the output feature map. By incorporating three types of attention mechanisms in different dimensions, enhancing adaptability in scale perception, spatial position, and multitasking aspects, the Dynamic Head significantly improves the expression capabilities of the detection head.

This paper presents several significant contributions:

(1) An enhanced CSPDarknet53 backbone network is introduced, leveraging the Swin Transformer architecture. This integration aims to better preserve contextual information.
(2) The original FPN structure is replaced with the RE-FPN, a lighter and more efficient residual feature fusion pyramid structure. Additionally, an SOD layer is integrated to bolster the adeptness of the model in detecting objects of various scales, particularly small objects.
(3) A Dynamic Head, equipped with multiple attention modules, is introduced to direct the model’s focus towards densely populated areas containing small objects. This facilitates the extraction of additional features from small objects. Experimental results demonstrate that our SRE-YOLOv8 model performs well and reaches a level of high accuracy in the specific dataset.

The subsequent sections of this paper are written as follows: the next section introduces relevant works on small object detection using CNNs and a Vision Transformer. Section 3 (Proposed Method) provides a detailed explanation of the specific improvement methods proposed in our work. Section 4 (Experiment) and Section 5 (Discussion) present the training results of the model and detailed analysis thereof. Finally, Section 6 (Conclusions) serves as the conclusion of this work.

## 2. Literature Review

### 2.1. SOD Based on CNN

In recent years, a multitude of researchers have dedicated substantial efforts to advancing small object detection, thereby augmenting the overall effectiveness of object detection in aerial drone imagery. For instance, Zhang et al. proposed the Super YOLO method for remote sensing imagery (RSI) object detection. This method integrates multimodal data and employs auxiliary super-resolution learning to achieve high-resolution detection of multi-scale targets [23]. Similarly, MAKTAB et al. tackled the challenge of SOD tasks in UAV imagery by introducing a CNN model that builds upon the Single-Shot Multi-Box Detector (SSD) as the baseline. Their approach enhances SOD performance by incorporating modules such as super-resolution, deconvolution, and an evolutionary way of feature fusion. However, its effectiveness in detecting objects in complex environments remains limited [24]. Additionally, Liu et al. enhanced YOLOv5 by introducing a Feature Enhancement (FE) Block, which generates features with different receptive fields. They combined this with spatial pyramid pooling to enhance the feature extraction capability. Nevertheless, this excessively deep feature extraction network led to the loss of its inherent feature information [25]. Qi et al., based on YOLOv7, made improvements through cropping, introducing attention mechanisms, reducing pooling kernel sizes, and refining downsampling methods. These enhancements focused on densely populated small target areas, thus boosting accuracy, but further improvements are needed in terms of lightweight design [26]. Kim et al. proposed a Channel Attention Efficient Pyramid YOLO (CAEP-YOLO) employing transpose convolution for upsampling to avoid significant information loss. However, this method faces challenges in extracting multi-scale features in complex backgrounds, resulting in poor performance in small object detection [27]. Wang et al. conducted optimizations on YOLOv8 by introducing a Feature Fusion Network Block (FFNB) designed to seamlessly integrate shallow and deep features. Building upon this, they added two detection heads, which significantly improved the detection performance in aerial imagery. However, this improvement led to a decrease in detection speed [28]. Conversely, Jawa et al. directed their efforts towards optimizing model efficiency. Their comprehensive approach included a range of strategies, such as clustering target box dimensions, pre-training the network for classification, training for multi-scale detection, and refining rules for filtering candidate boxes. These endeavors were aimed at enhancing the model’s ability to localize objects within the imagery [29]. The widespread application of YOLO series algorithms in small object detection research is evident. To further advance model enhancement, we chose YOLOv8 as the baseline model in this work.

### 2.2. Emergence of Vision Transformer

The Transformer architecture has garnered notable success in machine learning-based translation and the NLP domain, particularly in managing lengthy sequences and capturing extensive contextual information [30]. Building upon this foundation, researchers have extended the Transformer’s application into the realm of computer vision, yielding transformative models such as ViT [31], DETR [32], and the Swin Transformer, which plays an important role in this paper. These advancements have ushered in novel solutions to challenges within the visual domain. The inherent strength of the Transformer lies in its ability to directly compare features across all spatial and temporal positions, a trait that has translated into commendable performance in computer vision tasks [33]. In the realm of SOD, scholars have leveraged Transformer models to aggregate information from the entire image, thereby encapsulating global contextual information [34], and addressed the issue of dense targets by proposing a Deformable Transformer model, demonstrating good predictive performance for dense multitask learning. Ref. [35] tackled the problem of insufficient deep spatial information by designing a spatial interaction structure, improving edge information blur caused by occlusion. Ref. [36] proposed a fusion of convolutional neural networks and a Transformer in the backbone feature extraction network. By parallel use of region attention mechanism modules with the Swin Transformer, they extended information interaction within the window globally. In the pursuit of enhancing the accuracy of object detection within intricate backgrounds, the Swin Transformer stands as a noteworthy endeavor, marking a successful foray of the Transformer architecture into the domain of object detection. Starting from smaller image patches, the Swin Transformer progressively merges adjacent patches at deeper levels, constructing hierarchical feature representations. This structure enables the model to handle dense prediction tasks efficiently, achieving impressive object detection speed and accuracy in the MS COCO dataset [37]. Nonetheless, Transformer models, despite showcasing robust overall performance attributed to their global computation capabilities, may inadvertently overlook local information and demonstrate insensitivity towards small objects. This phenomenon can lead to a degradation in detection performance specifically concerning smaller objects [38]. To achieve better performance, we combine a CNN and a Transformer, integrating Swin Transformer modules with the YOLOv8 backbone network to bolster the model’s detection accuracy and robustness.

## 3. Proposed Method

**Figure 1:**
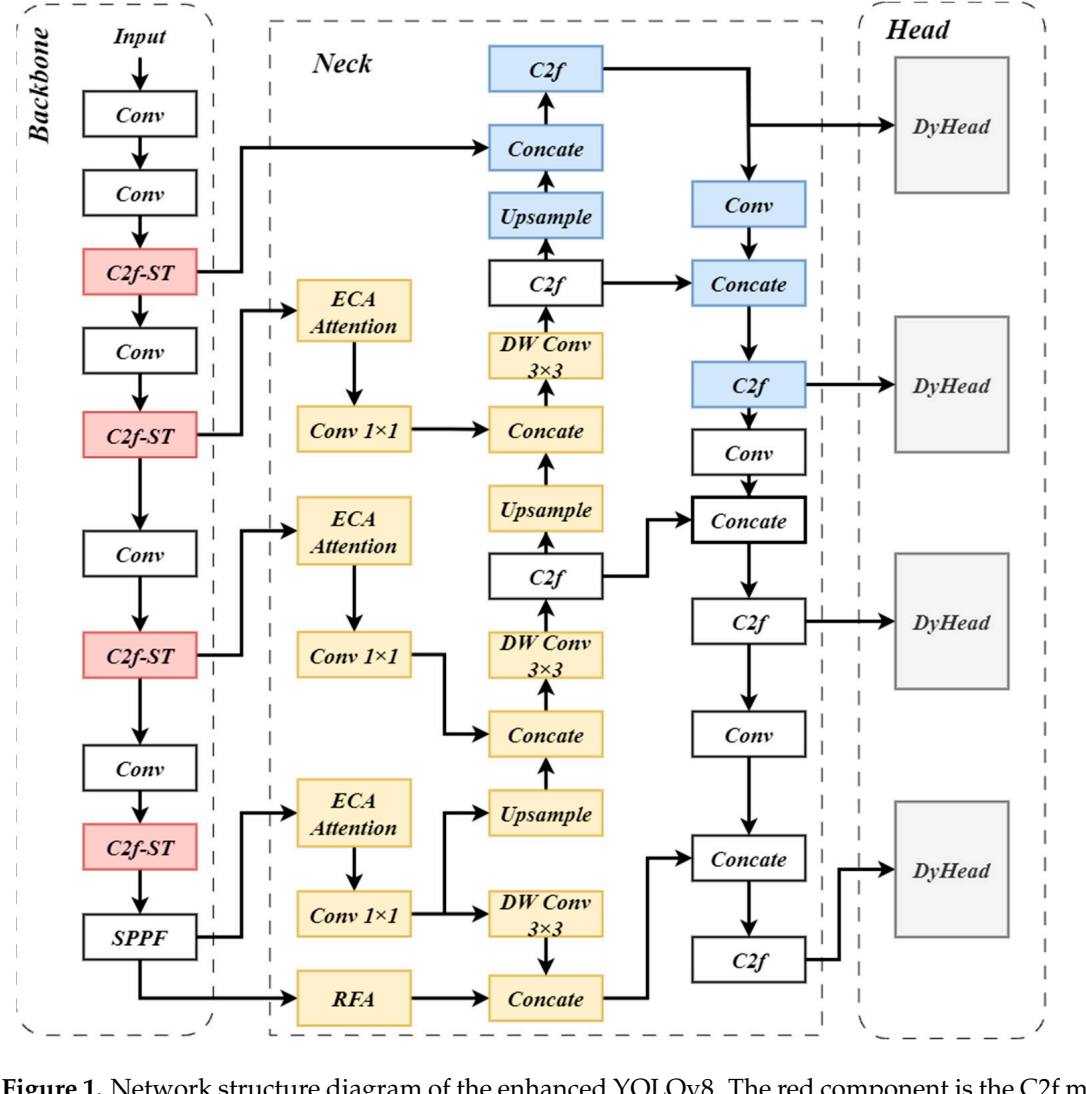
Network structure diagram of the enhanced YOLOv8. The red component is the C2f module of the backbone combined with the Swin Transformer; the yellow component is the integrated RE-FPN structure in the network; the blue component is the SOD small object detection layer; the light gray module is the improved detection head DyHead.

In Figure 1, we present the schematic of the SRE-YOLOv8 model for aerial drones. The enhancements to three components of the original YOLOv8 model are as follows: In the backbone section, we integrated the enhanced Swin Transformer module with the C2f (Cross-stage Partial Bottleneck with 2 convolutions) module and embedded it within the CSPDarknet53 network to augment feature extraction capabilities of the model. Next, we replaced the original FPN structure of the model with the lightweight residual feature fusion pyramid structure, RE-FPN. Additionally, within the neck section, we introduced a dedicated layer tailored for detecting small objects, enlarging the scales of feature maps utilized for predictions. This augmentation enhances the model’s ability to hone in on positional information crucial for accurate detection. Finally, we introduced the Dynamic Head as the detection head, employing three different channel-wise attention mechanisms to refine the model’s attention to key information of feature maps during prediction, thereby improving detection accuracy.

### 3.1. Improved Swin Transformer Module

To tackle the challenge of detail information loss during feature extraction in large-scale complex-scene UAV images by the YOLOv8 model, we introduced a novel integrated architecture, termed CSPLayer_2Conv-Swin Transformer (C2f-ST). Enhancing the network’s receptive field, this improved structure boosts efficiency, captured global information more effectively, and enriched contextual understanding.

**Figure 2:**
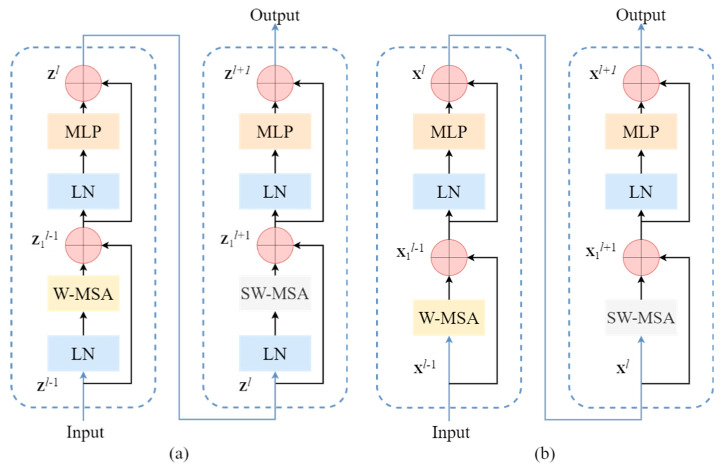
Modifications to the Swin Transformer module: (**a**) the original structural diagram; (**b**) the structural diagram after modifications.

The architecture of the Swin Transformer is visually depicted in Figure 2, showcasing its components, which include a Layer Normalization (LN) module, a window-based multi-head self-attention (W-MSA) module, a shift-window-based multi-head self-attention (SW-MSA) module, and a two-layer multi-layer perceptron (MLP) module featuring a Gaussian Error Linear Unit (GELU) non-linear layer. The LN module aids in seamlessly integrating the network, effectively preventing overfitting. Moreover, W-MSA and SW-MSA play pivotal roles in enabling the model to focus on pertinent information within adjacent windows and facilitate feature interactions across windows. Finally, the MLP module is instrumental in feature transformation and serves as the non-linear component of the residual connection mechanism.

The following equations elegantly represented the specific process of forward feature data transition from layer l to layer l+1:

(1) $$z_{1}^{l-1}=W\text{-}MSA\left(LN\left(z^{l-1}\right)\right)+z^{l-1}$$

(2) $$z^{l}=MLP\left(LN\left(z_{1}^{l-1}\right)\right)+z_{1}^{l-1}$$

(3) $$z_{1}^{l+1}=SW\text{-}MSA\left(LN\left(z^{l}\right)\right)+z^{l}$$

(4) $$z^{l+1}=MLP\left(LN\left(z_{1}^{l+1}\right)\right)+z_{1}^{l+1}$$

Here, $z^{l-1}$ represents the forward feature map for this module, and $z^{l+1}$ represents the backward feature map of this module. However, using LN in CNN may disrupt the learned sample features [39]. Therefore, to preserve the model’s generalization ability while protecting the CNN’s capability to learn sample features, we have removed the LN from the input part of the cascaded Swin Transformer, rendering it particularly well suited for integration within the base network. This adapted Swin Transformer module is visually depicted in Figure 2.

The enhanced process of forward feature data transition from entrance layer to exit layer can be succinctly expressed through the following equations:

(5) $$x_{1}^{l-1}=W\text{-}MSA\left(x^{l-1}\right)+x^{l-1}$$

(6) $$x^{l}=MLP\left(LN\left(x_{1}^{l-1}\right)\right)+x_{1}^{l-1}$$

(7) $$x_{1}^{l+1}=SW\text{-}MSA\left(x^{l}\right)+x^{l}$$

(8) $$x^{l+1}=MLP\left(LN\left(x_{1}^{l+1}\right)\right)+x_{1}^{l+1}$$

**Figure 3:**
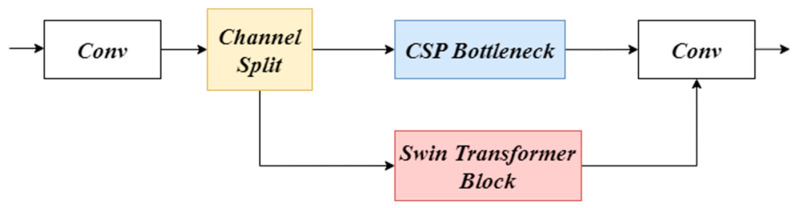
Structural diagram of the C2f module embedded with the improved Swin Transformer Block.

We inserted the improved Swin Transformer module into the C2f module to create a new C2f-ST module. The structural diagram of the C2f-ST module is shown in Figure 3. Following this, we replaced the original C2f modules in YOLOv8’s backbone network with the upgraded C2f-ST modules. By harnessing the distinctive attributes of the Swin Transformer, this adaptation enables more effective capture of information pertinent to small objects, consequently enhancing the transferability of learned features.

### 3.2. Lightweight Residual Feature Pyramid Network

**Figure 4:**
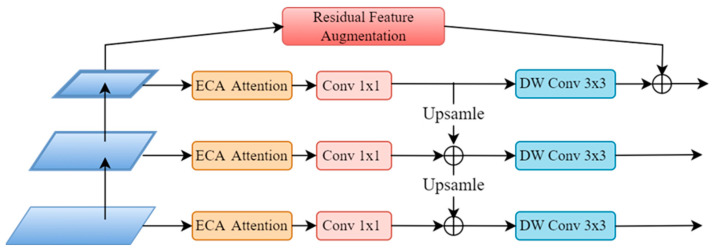
Lightweight residual feature fusion pyramid structure.

In deep neural networks, shallow features contain more spatial location information and fine-grained details, but they also include more noise. As the network deepens, semantic information increases in deep features, while the information about the object’s location and fine details gradually diminishes, and noise tends to decrease. This paper focuses on the lightweight design and improvement of the YOLOv8 algorithm’s neck network, specifically the feature pyramid network (FPN) based on feature fusion. The goal is to promote the flow and interaction of features at different depths. In addition, addressing the serious issue of spatial information loss caused by channel changes in high-level features within the FPN structure, a Residual Feature Augmentation (RFA) unit was innovatively assembled into the model architecture. The RFA module utilizes a residual branch to inject contextual information from different spatial locations, improving the feature representation of high-level features. Furthermore, a lightweight attention mechanism called the Efficient Channel Attention (ECA) module was inserted into each branch structure of the FPN to alleviate spatial information loss. Lastly, a 3×3 depthwise convolution (DW Conv) operation was applied to every feature map, resulting in the construction of a lightweight residual feature fusion pyramid structure denoted as RE-FPN, as illustrated in Figure 4. This design aims to enhance feature interaction, mitigate spatial information loss, and maintain a lightweight characteristic in the YOLOv8 algorithm’s FPN.

#### 3.2.1. Residual Feature Augmentation Module

The FPN structure, built upon inherent feature hierarchies, enhances object detection performance by propagating strong semantic features from higher levels to lower levels through feature fusion. However, as features are fused, they propagate in a top-down fashion, enabling lower-level features to benefit from the strong semantic information of higher levels. Nonetheless, there is a risk of information loss at the highest pyramid level due to channel reduction. To tackle this issue, this paper adopts adaptive pooling to extract diverse contextual information, thereby reducing information loss at the highest level of the feature pyramid through a residual enhancement approach. By introducing the Residual Feature Augmentation (RFA) module, which injects contextual information from different spatial positions using a residual branch, we aim to improve the feature representation of high-level features. This resulted in a more concise structure with reduced computational overhead.

**Figure 5:**
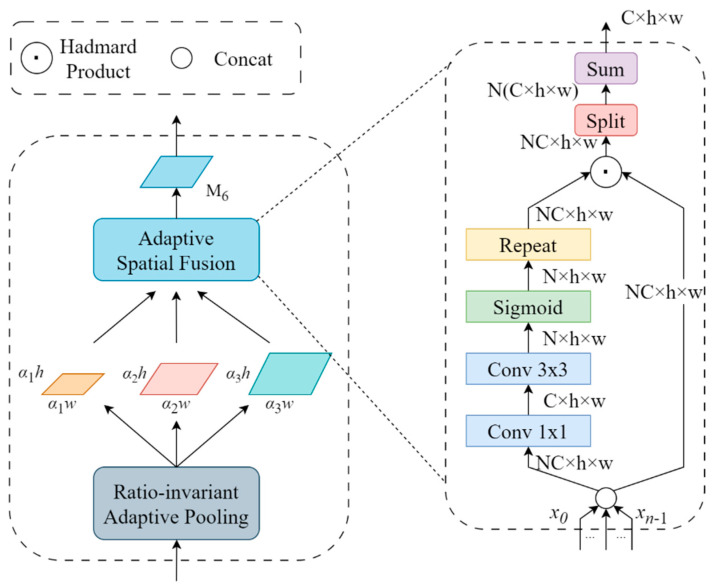
Residual Feature Augmentation structure diagram.

The RFA module effectively addressed spatial information loss in the feature maps extracted by the front network by integrating spatial contextual information, thereby enhancing the performance of the feature pyramid. The construction of the RFA module involved several specific steps: firstly, through scale-invariant adaptive pooling operations on a feature map S=h×w, multiple contextual features of different scales and depths (∂_1_×S, ∂_2_×S, …, ∂_n_×S) were generated. Subsequently, each contextual feature underwent a 1×1 convolution operation to diminish the dimension of channel feature. These processed features were then upsampled to scale S using bilinear interpolation, facilitating subsequent fusion operations. To address potential artifacts arising from interpolation, we introduced an Adaptive Spatial Fusion (ASF) module. This module dynamically combines contextual features in an adaptive manner, as opposed to a straightforward summation, mitigating the effects of interpolation-induced artifacts. By taking the upsampled features as input, producing a spatial weight map for every feature and aggregating contextual features into M using these weights, the ASF module imparted multi-scale contextual information. Figure 5 illustrates the structural diagram of the RFA module.

#### 3.2.2. Introduction of Lightweight Attention Mechanism

**Figure 6:**
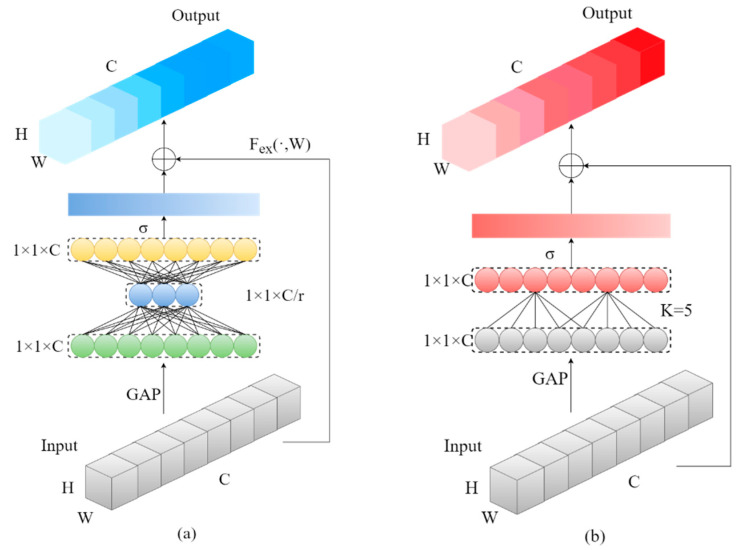
The ECA module achieves lightweight design by eliminating intermediate dimension reduction operations: (**a**) SE structure diagram; (**b**) ECA structure diagram.

Attention mechanisms originate from the study of human vision, enabling selective focus on informative regions while disregarding less relevant visual information [40]. Drawing inspiration from this, similar attention mechanisms have been introduced in deep learning. The lightweight attention mechanism, particularly the ECA (Efficient Channel Attention) module, represents an advancement of the channel attention model, building upon the SE (Squeeze-and-Excitation) attention mechanism [41]. This enhancement substantially reduces the network’s parameter count while mitigating the side effects linked to dimensionality reduction in the SE attention module. The structural diagrams of the SE attention module and the ECA attention module are depicted in Figure 6, respectively. Both attention mechanisms comprise three main steps: compression, excitation, and feature recalibration, detailed as follows.

Step 1: The compression operation utilizes GAP (Global Average Pooling) unit to compress the forward feature map in the spatial dimension, resulting in a 1×1×C real number sequence with global information. This addresses the issue of low information utilization between different layers in the network.

Step 2: The excitation operation corresponds to the function $F_{ex}(\cdot ,W)$ in the diagram and represents the difference between the ECA and the SE. The SE module employs two fully connected layers to reduce the number of feature channels C to 1/r, reducing computational load. In contrast, the ECA attention mechanism abandons the intermediate dimension reduction operation, choosing to utilize each channel and its K neighbors to obtain local cross-channel information. This approach circumvents information loss stemming from dimension reduction, thereby effectively capturing interactions among cross-channel information. After channel recovery to C, weights for each channel are obtained through an activation function.

Step 3: In the feature recalibration step, the weights obtained from the compression step are element-wise multiplied with the original features channel-wise, achieving feature recalibration.

### 3.3. Adding Small Object Detection Layer

Within the YOLOv8 framework, three distinct feature maps of varying sizes are employed to facilitate the detection of objects spanning a range of dimensions. Nonetheless, a significant challenge arises when objects within an image exhibit dimensions where either the width or height is less than 8 pixels, resulting in inadequate feature learning and consequent missed detections. Traditional strategies aim to rectify this by optimizing feature learning through image balance. However, in scenarios where the dataset is extensive, simple downsampling approaches may introduce a downsampling factor that is excessively large, leading to an unwarranted loss of information. Conversely, with a relatively small dataset, the network’s forward propagation requires storing numerous feature maps in memory. This could strain GPU resources, leading to memory overflow and hindering normal training and inference processes [42].

Hence, we augmented the model with a feature fusion layer and multi-scale feature extraction layer positioned atop the existing feature extraction layer, expanding it into a four-scale detection branch structure. The specific method involves adding the feature map from the 2nd layer of the backbone section to the feature fusion network. This helps the network better capture semantic information from shallow layers, thereby improving the detection accuracy for extremely small targets, as shown in Formula (9):

(9) P2=C3ConcatUpsampleP24neck,P24Backbone

**Figure 7:**
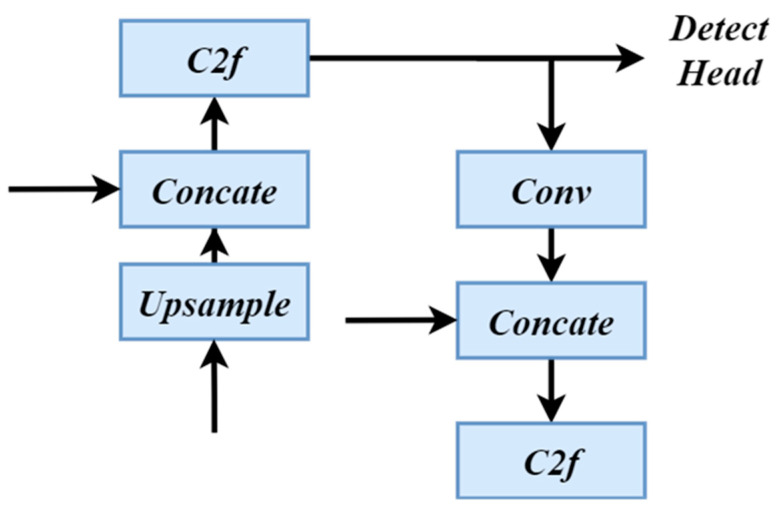
The structure diagram of SOD layer.

Here, the notation $P2/4_{neck}$ signifies that the dimension of the P2 layer in the neck is reduced by a factor of 4 compared to the input image size. Similarly, ${P2/4}_{backbone}$ represents that the size of the P2 layer in the backbone network is reduced by a factor of 4 relative to the input image size. Specifically, after two upsampling and concatenation operations, another upsample operation is performed to obtain a feature map with a larger size. The feature map, after upsampling and fusion of features at different scales, possesses stronger semantic information and finer spatial details, enabling better differentiation and detection of extremely small objects. Subsequently, the obtained feature map undergoes concatenation with the output of the second layer of the backbone network, further merging features of different scales. A C2f module is introduced to process the fused feature map, maintaining its size at 160×160, ultimately resulting in an ultra-small object detection layer. The structure diagram of the small object detection (SOD) layer is shown in Figure 7.

This approach enables the network to prioritize features at various scales, enhancing its adaptability to targets of varying sizes. Specifically, in the detection of extremely small objects, the fusion of multi-scale features aids the network in capturing finer details of targets. Moreover, the enhanced SOD layer utilizes the output of the feature map before the third upsample for concatenation. These enhancements improve the model’s detection performance concerning subtle textures, boundaries, and minor variations, facilitating more precise localization and recognition of targets in complex scenarios. Concurrently, it fortifies the model’s capacity to fuse multi-scale features, rendering it more adept at detecting targets of different sizes in unmanned aerial vehicle (UAV) capture scenarios, thus enhancing small object detection performance.

### 3.4. Dynamic Head

**Figure 8:**
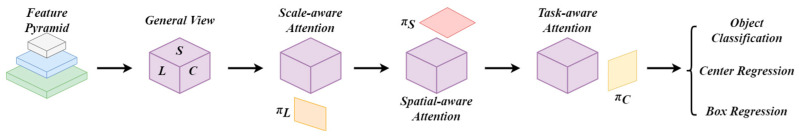
Dynamic Head overall structure diagram. $\pi_{L}$, $\pi_{S}$, $\pi_{C}$ represent attention mechanisms on three different dimensions, respectively.

In some scenarios, images captured by unmanned aerial vehicles (UAVs) often exhibit complex backgrounds, including dense small target data, potential occlusions, and unclear details. Therefore, having comprehensive perception capabilities in detection algorithms is crucial. YOLOv8 adopts a multi-level feature fusion detection head, separating classification and detection. However, due to the large perceptual field and relatively low resolution in aerial images, effectively detecting and locating small objects remains challenging. While traditional algorithms attempt to improve the detection head, they lack a unified perspective. Dynamic Head introduces a novel dynamic head framework, unifying the detection heads for various targets using an attention mechanism, as illustrated in Figure 8. Through harnessing attention mechanisms across scale-aware feature hierarchies, spatial positions for spatial awareness, and output channels closely related to task awareness, this approach markedly enhances the model’s expressive capabilities for target representation. This enhancement affords greater flexibility and precision in detecting small objects.

The scale-aware attention module intelligently fuses features of varying scales, prioritizing them based on their semantic significance, as shown in Formula (10):

(10) $$\pi_{L}\left(F\right)\cdot F=\sigma \left(f\left(\frac{1}{SC}\sum_{SC}F\right)\right)\cdot F$$

f(·) is an approximation of a linear function using a 1×1 convolution, denoted as $\sigma \left(x\right)=max(0,\;min(1,(x+1)/2))$.

The spatial-aware attention module begins by employing deformable convolution to acquire sparsity, following which it amalgamates cross-layer features situated at identical spatial positions, as delineated in Formula (11) [43]. Here, *K* denotes quantity of sparsely sampled positions, $p_{k}+\Delta p_{k}$ signifies autonomously learned spatial shift focused at the mobile position within a distinct region, and $\Delta m_{k}$ represents the independently learned significant scalar at position $p_{k}$. These parameters are all derived from the forward features of the middle layer *F*.

(11) $$\pi_{s}\left(F\right)\cdot F=\frac{1}{L}\cdot \sum_{l}^{L}\sum_{K=1}^{K}\omega_{l,k}\cdot F\left(l;p_{k}+\Delta p_{k};c\right)\cdot {\Delta m}_{k}$$

(12) $$\pi_{C}\left(F\right)\cdot F=\max\left(\alpha^{1}\left(F\right)\cdot F_{C}+\beta^{1}\left(F\right),\alpha^{2}\left(F\right)\cdot F_{C}+\beta^{2}\left(F\right)\right)$$

**Figure 9:**
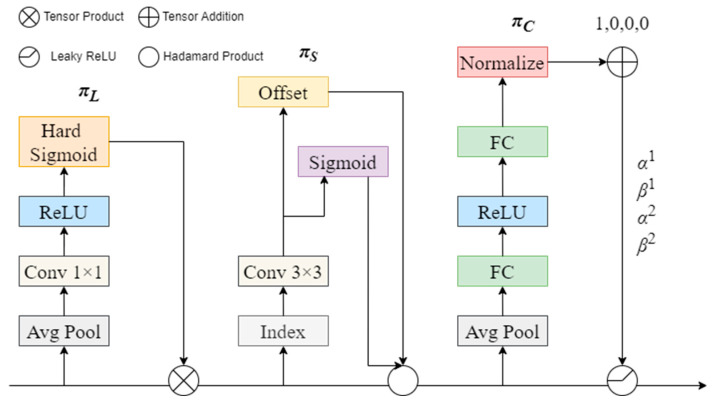
Detailed structural diagrams for the three attention mechanisms of Dynamic Head.

The task-aware attention module dynamically regulates the aperture of feature channels, thereby accommodating various tasks, as illustrated in Formula (12). Within the structural diagrams, the function $\theta (\cdot )=[\alpha^{1},\beta^{1}{,\alpha }^{2},\beta^{2}{]}^{T}$ is introduced to regulate the activation threshold. This function initially conducts global pooling to reduce dimensions across the L×S plane, followed by two cascaded fully connected layers, an NL module, and ultimately, the application of a shifted sigmoid function to normalize the output value to the range of [−1, 1] [44]. The detailed schematic diagrams of these three attention modules are shown in Figure 9.

## 4. Experiment

### 4.1. Dataset

**Figure 10:**
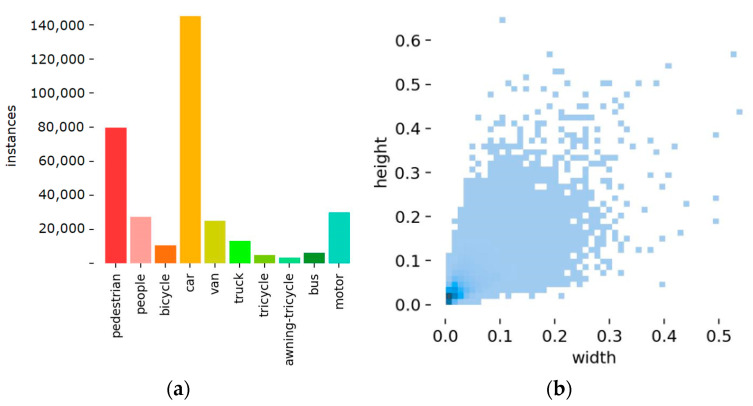
Dataset information features: (**a**) the distribution of the classes of the labels in VisDrone2021, distinguish different categories with colors; (**b**) bounding box size distribution heat map, the darker the color, the more labels of this size.

This paper employs the VisDrone2021 dataset for model training and evaluation. The dataset was collected by the AISKYEYE team. This dataset comprises images taken by a multitude of unmanned aerial vehicle cameras, encompassing a diverse array of locations (spanning 14 distinct cities across China, separated by thousands of kilometers), environments (encompassing both urban and rural settings), and objects (including pedestrians, vehicles, bicycles, etc.). Additionally, the dataset features scenes with varying densities, ranging from sparsely populated areas to densely crowded locales. For this experiment, a total of 7019 UAV aerial images were all used, covering pedestrians, people, cars, vans, buses, and so on, divided into ten categories. Among these, 6471 images were used for training and 548 images for validation. In addition, there are 1610 images for testing, and the labels of the test dataset will only be available for download during the annual challenge. The distribution of training data volume for each category and the size of labels in the training image set are shown in Figure 10. From the graph, it can be discerned that the number of labels varies across categories, with significant differences in data volume between corresponding categories. Additionally, most points in the label size distribution graph are concentrated in the bottom left corner, with a few points scattered in the middle and upper right corners. This indicates that VisDrone2021 exhibits a large number of small objects and some medium-sized objects, showing diverse object sizes that align with the context and problems addressed in this paper.

### 4.2. Experimental Environment and Parameter Settings

The software and hardware of the experimental training environment were as follows: an i9-13900K processor served as the computational backbone. All models underwent training and testing on an NVIDIA RTX 4090 GPU. The program was developed within the Windows 10 operating system, utilizing the PyTorch framework and CUDA Toolkit 11.2 for enhanced computational efficiency.

Other hyperparameter settings for training are shown in Table 1.

Furthermore, conventional optimization strategies such as warm-up training, cosine annealing, etc., were judiciously employed to enhance model performance and convergence.

### 4.3. Evaluation Metrics

Following the completion of model training, the trained weights were utilized to validate the model, subjecting it to evaluation from multiple angles. The model’s performance underwent rigorous assessment using metrics including precision, recall, and mean average precision (mAP). Precision, denoted as the ratio of true positives to all detected objects, and recall, calculated as the ratio of correctly detected objects to the total number of labeled objects, served as pivotal indicators. The exact formulas for these calculations are as follows:

(13) $$P=\frac{TP}{TP+FP}\times 100\%$$

(14) $$R=\frac{TP}{TP+FN}\times 100\%$$

In the provided formulas, *TP* represents the count of correctly detected objects by the model, while *FP* signifies the tally of objects erroneously detected by the model, and *FN* represents the number of correctly labeled objects that the model fails to detect. The area under the precision–recall curve corresponds to the average precision (*AP*) for the given category, with its calculation depicted as Formula (15):

(15) $$AP=\int_{0}^{1}P\left(R\right)dR$$

(16) $$mAP=\frac{1}{c}\sum_{i=1}^{c}AP_{i}$$

In the formula, c is the number of classes in a multi-class detection task, and ${AP}_{i}$ is the average precision for the i-th class object. The average of all categories’ average precision (*AP*) values in Formula (16) is referred to as mAP (mean average precision).

### 4.4. Experiment Result

**Figure 11:**
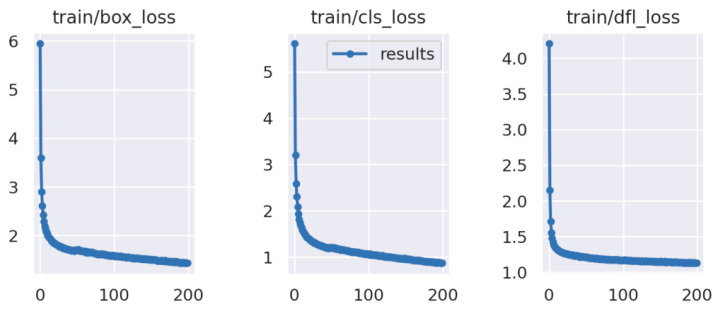
Training curve of our proposed model.

Figure 11 illustrates the variations in common metrics during the training process of our proposed improved algorithm. Based on the YOLOv8 model, three loss functions were employed during training to update model parameters through backpropagation, optimizing model performance. “box_loss” represents the localization loss, aiding in ensuring accurate object localization by the model. “dfl_loss” is the confidence loss, calculating the CIOU between the predicted bounding box and GT box. “cls_loss” is the classification loss, evaluating the model’s accuracy in predicting object categories. From the graph, it can be observed that the model starts to converge around 30 epochs, indicating that our improvements did not affect the convergence speed of the model. By the time it reaches 200 epochs, all loss functions have decreased to their limits, suggesting that the model has completed training.

**Figure 12:**
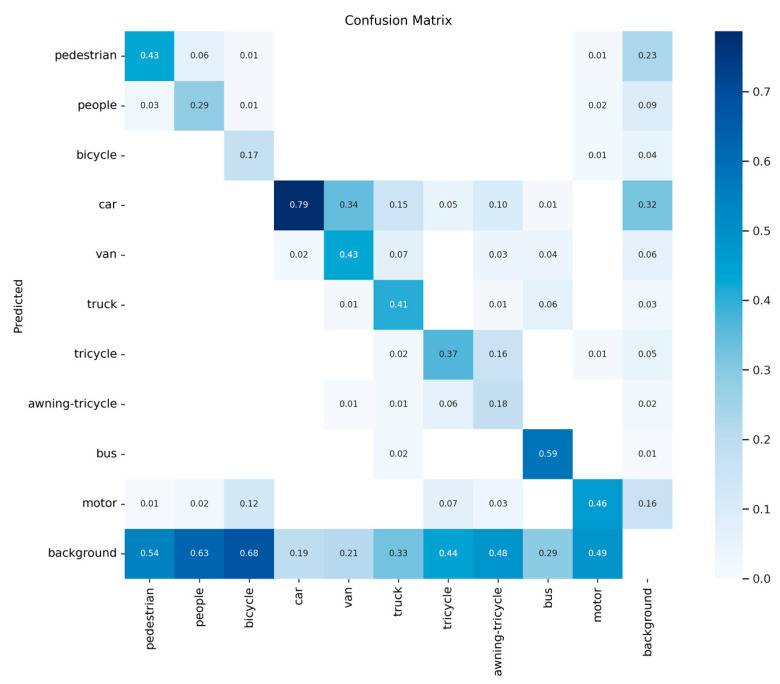
The confusion matrix of the proposed work on the VisDrone2021 dataset.

The confusion matrix of the improved algorithm is shown in Figure 12. The confusion matrix serves as a visual representation of the classification results for each category. Each row within the matrix corresponds to the predicted category, while each column represents the actual category. The data on the diagonal indicate the proportion of correctly classified categories. However, the *FN* (false negative) values for the tricycle, people, and awning-tricycle categories are relatively high. This implies that targets belonging to these three categories are often missed during the detection process. The main reason for this is that the count of objects in these categories is significantly lower than in others, leading to limited feature extraction due to a lack of training samples. The *TP* (true positive) values for the pedestrian, car, and motor categories are relatively high, indicating that the model performs well when detecting targets of these three categories.

## 5. Discussion

### 5.1. Comparative Tests with YOLOv8

Table 2 illustrates the comparative performance between our SRE-YOLOv8 and the original YOLOv8 baseline model. Our model exhibits notable enhancements in both individual category accuracy and mAP@0.5 when compared to the baseline. Specifically, there are approximately 10% improvements in AP for pedestrian, car, tricycle, awing-tricycle, and motor categories. Additionally, the mean average precision (mAP) across all categories increased by 9.2%. These results signify a significant advancement in the accuracy of SOD in aerial drone images, ultimately leading to an improvement in overall detection performance.

**Figure 13:**
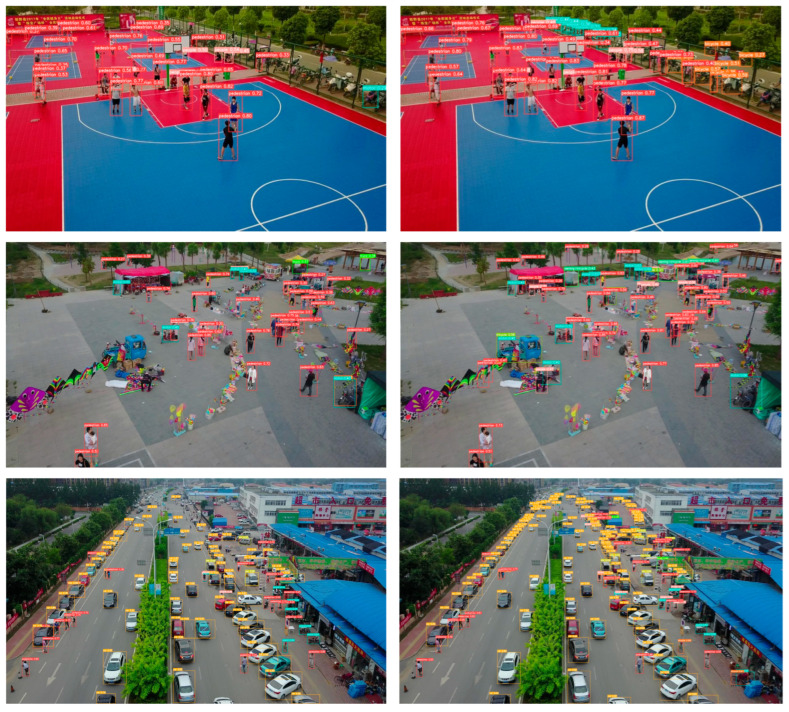
Representative detection results of proposed work. Different colors show different classes.tar.

To visually elucidate the detection prowess of the enhanced algorithm, a subset of representative images was handpicked from the test dataset, as depicted in Figure 13. The trio of images on the left exhibit the detection outcomes generated by the original YOLOv8 model, whereas the trio of images on the right showcase the detection outcomes produced by our SRE-YOLOv8 proposed in this study. It can be observed that the improved model can detect more small targets, demonstrating the good recognition capability for dense, low-resolution small targets in UAV aerial images.

### 5.2. Comparative Analysis of C2f-ST Module

This paper introduces the improved Swin Transformer into the C2f module, thereby constructing the C2f-ST module, which is seamlessly integrated into the CSPDarknet53 backbone network, amplifying the model’s feature extraction prowess. To comprehensively validate the efficacy and superiority of the C2f-ST module, we meticulously selected and conducted comparative experiments with various modules, namely C2f, C2f_MixConv, C2f_DefConv, and C2f_CrossConv modules. Keeping all other network structures and training parameters constant, we embedded the C2f-ST module into the YOLOv8 base model at identical positions, sequentially incorporating the aforementioned five enhanced C2f modules into the model. The experimental findings are meticulously detailed in Table 3, where FLOPs denote the total computational cost of the model.

The experimental results indicate that, although C2f-ST has a slight increase in training load, its accuracy, recall rate, and average precision are higher than other feature extraction modules, demonstrating the effectiveness of the C2f-ST module in terms of accuracy.

### 5.3. Comparative Analysis of RE-FPN Structure

To validate the effectiveness of the lightweight residual feature pyramid network structure (RE-FPN), we used the YOLOv8 algorithm with FPN as the baseline. We compared the detection performance of YOLOv8 algorithms using different feature fusion structures, including FPN, SE-FPN, BiFPN [45], and RE-FPN. Among them, SE-FPN is the residual feature pyramid network using an SE attention mechanism. The experimental results are shown in Table 4.

The experimental data in the above table show that after introducing SE-FPN, BiFPN, and RE-FPN structures into the model, the model’s parameters and detection accuracy were improved to varying degrees, but at the same time the inference speed of the model was reduced. Among them, the algorithm using the BiFPN structure has the highest detection accuracy, but due to the excessive number of parameters in the BiFPN structure, this leads to a notable decrease in inference speed. Conversely, the RE-FPN structure also markedly enhances the model’s detection accuracy. Due to its lightweight residual structure, it does not lead to a surge in parameters, ensuring the inference speed of the detection model.

### 5.4. Ablation Experiment

To investigate the individual impact of each module on detection performance, we conducted a series of ablation experiments using the test dataset of VisDrone2021. We utilized YOLOv8 as the base model to maintain fairness and accuracy, employing identical training data and parameter settings across all experiments. Results presented in Table 5 indicate the utilization of each method to enhance the model, denoted by “√”.

From Table 5, it can be observed that each enhancement module contributes to a certain improvement in the detection accuracy. The impact of the C2f-ST module is particularly significant; its introduction resulted in a substantial 5.8% increase in detection mAP compared to the initial model. However, the introduction of the module increased the model parameters, leading to a decrease in inference speed. This issue can be addressed by slightly improving the hardware performance of the deployment device, making it acceptable. The introduction of the RE-FPN and the SOD layer strengthened the neck part, enhancing its feature fusion capability and further boosting detection performance. Especially benefiting from the lightweight design of the RE-FPN and its efficient feature propagation capability, the entire model can achieve a satisfactory increase in average precision without a significant loss in detection speed. The Dynamic Head detection head utilizes three different-dimensional attention mechanisms, requiring almost no additional parameters, enabling the model to better capture spatial and positional information in the feature map, thereby enhancing its generalization ability. In the end, the inference speed of the model is effectively reduced, but the detection accuracy is significantly improved, with a mAP increase of 9.2%.

### 5.5. Comparative Experiments with Other Models

To further quantitatively evaluate the detection performance of the proposed algorithm, comprehensive detection tests were conducted on the VisDrone2021 dataset alongside other improved algorithms. The resulting detection accuracy for each category and the average detection accuracy are meticulously presented in Table 6.

Analysis of the table reveals the enhanced algorithm introduced in this paper attained the highest average accuracy among all the compared algorithms, surpassing them in six out of the ten categories. Specifically, we tested the performance of the latest model in the YOLO series on this dataset. Our model outperforms the YOLOv9-c model in both the recognition accuracy and average precision of most categories. Due to reasons such as network depth and the small size and dense arrangement of some objects, the accuracy of the proposed algorithm in detecting certain categories was slightly lower, but the difference from other algorithms wase not significant. Overall, this algorithm outperforms other methods, validating the effectiveness of the improvements.

## 6. Conclusions

We presented SRE-YOLOv8, a novel multi-scale feature fusion object detection algorithm, harnessing an enhanced Swin Transformer alongside a lightweight residual feature pyramid network (RE-FPN). Initially, we integrated the Swin Transformer with YOLOv8 via the C2f module, introducing an enhanced CSPDarknet53 structure designed to more efficiently capture both global and local semantic information. Subsequently, we refined the feature pyramid network within the neck of the model by integrating the RFA module and the ECA attention mechanism, thus forming the RE-FPN structure. This structure aims to enhance the fusion of shallow and deep semantic features, resulting in a feature representation abundant in both positional and semantic information. Moreover, we incorporated a dedicated SOD layer within the neck, leveraging larger-scale feature maps to reinforce the ability of the model to detect objects across multiple scales. Additionally, we employed a Dynamic Head as the detection head of the model to emphasize its attention on small targets. The experiments indicated that these improvements lead to a 9.2% increase in accuracy. In future research, we intend to explore methods to enhance small object detection accuracy while mitigating computational complexity, maintaining model accuracy, and accelerating inference, to cater to applications in drone aerial scenarios with constrained computing resources. 

## Figures and Tables

**Table 1:** Hyperparameter setting.

Hyperparameter Item	Value
Optimizer	SGD
Momentum Parameter	0.941
Weight Decay Coefficient	0.0004
Initial Learning Rate	0.011
Epoch	200
Image Size	640 × 640
Batch Size	32

**Table 2:** Accuracy comparison between baseline and proposed work.

Method	Ped	Peo	Bic	Car	Van	Tru	Tri	Awi	Bus	Mot	mAP@0.5 (%)
YOLOv8	40.3	23.1	10.2	67.4	35.1	26.9	11.7	10.1	31.3	32.1	29.3
SRE-YOLOv8 (Ours)	50.2	36.6	14.1	79.5	36.5	31.9	23.3	19.6	45.5	47.7	38.5

**Table 3:** Performance comparison table of different feature extraction module.

Feature Extraction Module	FLOPs (G)	Precision (%)	Recall (%)	*mAP*
C2f	15.8	38.6	28.1	29.3
C2f_MixConv	28.5	37.5	31.4	33.5
C2f_DefConv	28.1	38.1	30.6	34.6
C2f_CrossConv	27.9	37.3	29.1	33.9
C2f-ST	27.4	39.5	32.6	35.1

**Table 4:** Comparison of different feature pyramid modules.

Feature Fusion Structures	Parameter (M)	*mAP* (%)	FPS
FPN	33.5	29.3	59
SE-FPN	41.9	36.7	39
BiFPN	42.1	36.9	42
RE-FPN	38.6	36.1	54

**Table 5:** The experiment results of ablation study.

Improvement Method		Experiment							
		1	2	3	4	5	6	7	8
C2f-ST		-	√	-	-	-	√	√	√
RE-FPN		-	-	√	-	-	√	√	√
Small Object Detection Layer		-	-	-	√	-	-	√	√
Dynamic Head		-	-	-	-	√	-	-	√
Evaluation Indicator	*mAP* (%)	29.3	34.1 (+4.8)	31.2 (+1.9)	29.6 (+0.3)	31.0 (+1.7)	36.2 (+5.9)	38.1 (+7.8)	38.5 (+8.2)
	*AP-S* (%)	17.4	20.5 (+3.1)	18.5(+1.1)	17.9(+0.5)	18.1(+0.7)	21.5(+4.1)	21.9(+4.5)	22.3 (+4.9)
	Precision (%)	38.6	39.5 (+0.9)	38.2 (−0.4)	40.0 (+1.4)	38.9 (+0.3)	39.7 (+1.1)	40.5 (+1.9)	41.3 (+2.7)
	Recall (%)	28.1	32.6 (+4.5)	29.4 (+1.3)	29.6 (+1.5)	28.8 (+0.7)	33.1 (+5.0)	34.1 (+6.0)	34.9 (+6.8)
	FPS	59	55	58	56	57	54	53	53

**Table 6:** Accuracy comparison experiments with other methods.

Model	Ped	Peo	Bic	Car	Van	Tru	Tri	Awi	Bus	Mot	mAP@0.5 (%)
CenterNet [46]	21.1	25.1	4.3	57.5	15.1	15.0	12.0	5.3	23.7	16.4	19.5
Fcos [47]	34.6	26.6	7.7	69.3	28.4	24.0	13.8	6.7	28.3	33.4	27.3
Tridentnets [48]	27.9	24.1	7.4	73	35.1	27.8	17.9	8.4	46.3	33.3	30.3
ATSS [49]	35.8	21.9	9.6	73.3	34.7	28.1	18.1	10.2	46.3	34.9	31.3
VFNet [50]	38.9	31.6	11.3	72.4	34.8	28.5	19.3	9.5	43.4	37.0	32.7
PP-PicoDet-L [51]	40.2	35.3	12.8	75.6	35.4	29.3	21.1	12.1	44.3	36.3	34.2
YOLOv9-c [52]	49.1	35.4	14.8	78.5	36.8	30.5	23.6	19.2	44.6	47.1	38.1
SRE-YOLOv8(Ours)	50.2	36.6	14.1	79.5	36.5	31.9	23.3	19.6	45.5	47.7	38.5

## Data Availability

Data sharing is not applicable to this article.

## Abbreviations

The following abbreviations were used in this manuscript:

UAVs	Unmanned aerial vehicles
SOD	Small object detection
RE-FPN	Residual Feature Augmentation
C2f	Cross-stage Partial Bottleneck with 2 convolutions
DW Conv	Depthwise convolution
LN	Layer Normalization
GELU	Gaussian Error Linear Unit
MLP	Multi-layer perceptron
W-MSA	Window Multi-head Self-Attention
SW-MSA	Shifted Window Multi-Head Self-Attention
ASF	Adaptive Spatial Fusion
GAP	Global Average Pooling
mAP	Mean average precision
AP-S	Average precision for small objects
